# Antimicrobial resistance patterns of *Escherichia coli* isolated from raw cow milk and, from clinical specimens in a tertiary institution, Uganda – A cross sectional study

**DOI:** 10.1371/journal.pone.0321341

**Published:** 2026-07-16

**Authors:** Jacob Michael Othieno, Anastacia Sebbowa Nabyonga, Abel Wembabazi, Brian Martin Odhiambo, Winnie Nalwanga, Amusa Wamawobe, Sabrina Bakeera-Kitaka, Beatrice Achan

**Affiliations:** 1 School of Medicine, College of Health Sciences, Makerere University, Kampala, Uganda; 2 School of Dentistry, College of Health Sciences, Makerere University, Kampala, Uganda; 3 Department of Paediatrics and Child Health, School of Medicine, Makerere University, Kampala, Uganda; 4 Department of Medical Microbiology, School of Biomedical Sciences, College of Health Sciences, Makerere University, Kampala, Uganda; Fayetteville State University, UNITED STATES OF AMERICA

## Abstract

**Background:**

Inappropriate antibiotic use in animal husbandry contributes to the emergence of resistant bacteria, which may enter the human population through the food chain.

**Objective:**

To compare antimicrobial resistance (AMR) patterns of *Escherichia coli* isolated from raw cow milk in Kawempe Division, Kampala, with those from clinical specimens processed at Makerere University Laboratory, Kampala, Uganda.

**Methodology:**

A cross-sectional study from January to June 2024 on 40 *E. coli* isolates from 124 raw cow milk samples, alongside 40 clinical *E. coli* isolates was conducted. Antibiotic susceptibility was tested using the Kirby Bauer disk diffusion method against 11 antibiotics. Differences in resistance between milk and clinical isolates were assessed using chi-square analysis.

**Results:**

Of the 124 milk samples (77 from cans, 47 from packs), *E. coli* was isolated from 40/124 (32.26%), with most 39/124 (31.45%) from cans and only 1/124 (0.81%) from a pack. Antibiotic resistance profiles of *E. coli* isolates (n = 40) from milk samples showed the highest resistance to ampicillin (32.5%), followed by cotrimoxazole (22.5%), ceftriaxone (5%), Cefotaxime; (5%) and, cefuroxime (5%). Multidrug resistance (MDR) and extended-spectrum β-lactamase (ESBL) production were detected in 5% milk isolates. Clinical isolates; n = 40; exhibited higher resistance to cotrimoxazole (95%), ampicillin (87.5%), cefuroxime (72.5%), ceftriaxone (70%), ceftazidime (57.5%), ciprofloxacin (55%), and cefotaxime (52.5%), with lower resistance to amoxicillin–clavulanate (27.5%), chloramphenicol (25%), and gentamicin (22.5%). Emerging resistance to imipenem was observed (20%). MDR and ESBL prevalence among clinical isolates were 85% and 70%, respectively. Resistance was significantly higher in clinical isolates than in milk isolates (p < 0.001).

**Conclusion:**

This study shows a much higher burden of resistance, MDR, and ESBL production in clinical than milk-derived *Escherichia coli*. At the same time, the similar resistance patterns observed for commonly used antibiotics such as ampicillin and cotrimoxazole suggest a possible overlap between human and animal reservoirs. These findings highlight the need for context-specific antimicrobial stewardship strategies in both the health and diary sector.

## Introduction

### Background

Antimicrobial resistance (AMR) is a global health concern claiming over 700,000 lives annually [1]. Without effective control measures, deaths are projected to rise to 10 million per year, with an estimated global economic loss of over US $100 trillion annually, and increased health care costs of US$ 1 trillion by 2050 [2,3].The World Bank also projects that AMR could reduce the global annual gross domestic product (GDP) by up to US$ 3.4 trillion annually by 2030 [4]. This growing crisis signals the onset of a post-antibiotic era, where routine infections become impossible or difficult to treat, in both humans and animals [5].

One of the key drivers of AMR is the widespread misuse and overuse of antibiotics in food-producing animals [6]. In many cases, antibiotics are not only used for treating infections but also routinely administered as growth promoters to boost productivity in animals [7,8]. The continued and unregulated use of these drugs creates constant selection pressure on microorganisms leading to the emergence and spread of drug-resistant bacterial strains, particularly in developing countries where enforcement of antibiotic use policies remains weak, [9,10]. Livestock, including dairy cattle, serve as significant reservoirs of these resistant pathogens [11,12], which may be transmitted to humans through direct contact [13], environmental exposure [14], or consumption of contaminated animal products like raw milk [15,16].

In Uganda and other low-income settings, the burden of foodborne illnesses has risen rapidly due to poor sanitation, lack of food safety enforcement, and unhygienic handling of animal products [6,17]. Raw cow milk, a widely consumed animal food product in many households in Uganda, is susceptible to fecal contamination during milking, handling, and storage [18]. Its high nutritional content makes it an ideal medium for bacterial growth, including antimicrobial-resistant *Escherichia coli* (*E. coli*) [19].

*E. coli* is a Gram-negative bacterium that normally inhabits the gastrointestinal tract of humans and animals [20]. However, due to mutations, there has been emergence pathogenic strains such as Enteropathogenic *E. coli* (EPEC), Enterotoxigenic *E. coli* (ETEC), Enteroinvasive *E. coli* (EIEC), Enterohemorrhagic *E. coli* (EHEC), Enteroaggregative *E. coli* (EAEC), and Diffusely Adherent *E. coli* (DAEC), [21] which have acquired virulence factors through genetic mechanisms, enabling them to cause serious diseases like diarrhoeal illnesses, hemorrhagic colitis, and hemolytic uremic syndrome [22].

Pathogenic *E. coli* strains not only pose serious public health risks but also present a high economic burden, leading to increased healthcare costs, longer hospital stays, reduced productivity, and trade restrictions on dairy products [23]. In addition to their disease causing potential, *E. coli* also has the ability to acquire and transfer antibiotic resistance genes by horizontal gene transfer (HGT) to other non-resistant bacteria through conjugation, transformation or transduction [24]. This capability allows *E. coli* to serve as both a reservoir and a vehicle for the spread of resistance to other bacteria in the environment, animals, and humans [25]. Due to this, *E. coli* is widely used as an indicator organism in AMR surveillance systems worldwide [26].

Despite its critical role in AMR transmission between humans, animals and the environment, limited research has been conducted in Uganda to assess the antimicrobial resistance profiles of *E. coli* from raw milk and compare them with clinical isolates. Understanding this linkage is essential for identifying zoonotic transmission pathways, informing antibiotic stewardship, and strengthening food safety policies using the One Health approach. This study compared the antimicrobial resistance profiles of *E. coli* isolates from raw cow milk in Kawempe Division, Kampala, with those from clinical specimens at Makerere University. Comparing resistance patterns across these sources therefore provided insight into differences in antimicrobial selection pressure and potential overlap in resistance mechanisms, which is essential for informing integrated antimicrobial resistance surveillance and control strategies in Uganda’s health and diary sector.

## Materials and methods

### Study design and site

A cross-sectional study was conducted between January and June 2024 in Kawempe Division, Kampala, Uganda due to its large and socio-economically diverse population, increasing its public relevance for investigating *E. coli* contamination and AMR. Raw cow milk samples were collected from 10 milk collection sites within the division, selected based on high customer turnover, representation of both informal and formal milk vendors, accessibility and geographical spread across the division to ensure diversity. Samples were obtained from milk cans and sealed packs in these sites. *E. coli* clinical isolates were obtained from the archived samples in the repository of Department of Medical Microbiology, Makerere University.

### Sample size estimation and sampling

The sample size for the milk samples was determined using the Kish and Leslie method of 1965, n= (Z²pq)/d² where Z was the standard normal value at 95% confidence (1.96), p was the estimated prevalence of *E. coli* contamination (44.57%), q = 1-p (55.43%) and d as the margin of error (0.05). The prevalence value of 44.57% was adopted from a previous study conducted among raw cow milk samples in Uganda [27] which provided a more contextually relevant estimate for the present-day population. A sample size of 380 milk samples was calculated. However, due to resource limitations, only 124 milk samples were analyzed. A mixed sampling strategy was used due to the dual nature of study samples (raw cow milk and clinical isolates). A purposive sampling technique was used because raw cow milk handling and distribution in the study were largely informal and heterogenous making probability-based sampling impractical. Purposive selection ensured inclusion of sites most relevant to public exposure and food borne transmission risk. A simple random selection of 40 clinical *E. coli* isolates was made from all the archived *E. coli* isolates between January and June 2024 in the Clinical Microbiology Laboratory. The *E. coli* isolates were obtained from pleural fluid [3] urine [18], blood [7], sputum [4], pus aspirates [1], vaginal swab [1], and pus swab [6] samples from patients aged 14–86 years. Random selection was used to minimize selection bias and ensure representativeness of all routine clinical infections. The clinical isolates were used to compare the resistance patterns of *E. coli* with those from milk samples. Although isolates from these milk and clinical sources were not epidemiologically linked at the individual level, they exist within interconnected human, animal, and environmental systems where antimicrobial resistance determinants may circulate. Foodborne exposure, environmental contamination, and indirect human-animal interactions provide pathways through which resistant bacteria and resistance genes can move across sectors hence the basis for the comparison.

### Eligibility criteria

#### Inclusion criteria.

The study included:

- Milk samples from milk shops (cans or packs) whose attendants consented to provide the milk samples.
- Archived *E. coli* isolates whose demographics were complete and properly labelled.

#### Exclusion criteria.

The study excluded:

- Milk samples from shops whose attendants were not willing to consent to the study
- All clinical samples whose demographics were incomplete or mislabeled and damaged milk samples during transportation.

### Sample collection and transportation

A total of 124 raw milk samples, each 1 mL were collected from cans and packs from milk shops and in sterile containers from Kawempe Division, Kampala, Uganda. The milk samples were collected aseptically by using methods that prevent contaminations such as gloved hands, sterile wide mouth falcon tubes, and closing the tubes as soon as the milk is collected. The collected milk samples were labeled and packed in a secondary container and transported with an ice box to the Microbiology Department laboratory at Makerere University, Kampala-Uganda with the accompanying laboratory request forms.

The archived *E. coli* isolates from the clinical specimens, originally collected for routine diagnostic purposes, were retrieved from the Microbiology Clinical Laboratory repository, where they were stored in glycerol stocks at −80 °C. Isolates collected between January and June, 2024 were identified using laboratory records, and only those with complete demographics and correct labeling were selected for the study

### Laboratory procedures

#### Isolation and characterization of *E. coli*.

Raw milk samples were processed immediately upon arrival. One milliliter of thoroughly mixed raw milk was aseptically inoculated to 9 mL of sterile nutrient broth and incubated overnight at 37˚C. After enrichment, 100 µL of the broth culture was onto sub-cultured on sterile MacConkey agar plate for isolation of Gram-negative bacteria. Presumptive *E. coli* colonies (pink, lactose fermenting) were subjected to a standard biochemical identification panel consisting of Tripple Sugar Iron (TSI), Sulfide Indole Motility (SIM), Citrate and Urease) for confirmation of *E. coli*. All the biochemical tests were performed and interpreted according to the CLSI standards (2024), and standard microbiology procedures. Confirmed isolates were preserved in 20% glycerol stocks at −80˚C until antimicrobial susceptibility testing was done.

#### Antimicrobial susceptibility testing.

Antimicrobial susceptibility testing for all *E. coli* isolates from milk and clinical specimens was performed using the Kirby-Bauer disk diffusion method, following the CLSI guidelines, 2024. Prior to testing, both milk-derived and clinical *E. coli* isolates preserved in glycerol stocks were aseptically thawed and sub-cultured on tryptic soy broth + glycerol to obtain fresh pure colonies. Fresh *E. coli* colonies were emulsified in sterile saline, and then standardized to the 0.5 McFarland turbidity standard to ensure a uniform bacterial density for testing. Each standardized suspension was spread evenly onto Mueller-Hinton Agar (MHA) to create a continuous lawn of bacterial growth. Commercial antibiotic discs were then placed on the surface of the inoculated MHA agar and incubated at 37°C for 24 hours. Zone diameters were measured in millimeters and interpreted as susceptible, intermediate, or resistant according to CLSI breakpoints (Table 1).

**Table 1:** CLSI standards: Antimicrobial concentrations and interpretation breakpoints for various antibiotic agents used in this study to interpret results.

		Breaking Point (mm)		
ANTIMICROBIAL AGENT	Disc Drug Concentration	Sensitive	Intermediate	Resistant
(Code)	(µg)	(S)	(I)	(R)
CRO (Ceftriaxone)	30	≥23	20-22	≤19
AUG/AMC (Augmentin/ Amoxycillin/ Clavulanic Acid)	20.0/10.0	≥18	14-17	≤13
CTX (Cefotaxime)	30	≥26	23-25	≤22
CAZ (Ceftazidime)	30	≥21	18-20	≤17
CXM (Cefuroxime)	30	≥18	15-17	≤14
AMP (Ampicillin)	10	≥17	14-16	≤13
CIP (Ciprofloxacin)	5	≥26	22-25	≤21
IMP (Imipenem)	10	≥23	20-22	≤19
C (Chloramphenicol)	30	≥18	13-17	≤12
CN (Gentamicin)	10	≥18	15-17	≤14
SXT/COT (Cotrimoxazole)	1.25/23.75	≥16	11.0-15	≤10

The breaking points (S = sensitive, I = intermediate, and R = resistant) of the antibiotics at particular disc concentrations.

### ESBL screening and confirmation

Isolates were initially screened for possible ESBL production based on reduced susceptibility to third generation cephalosporins (Cefotaxime, ceftazidime, and ceftriaxone). Any isolate that showed decreased zone diameters or non-susceptibility to these agents was considered potential ESBL producers. Screen-positive isolates were confirmed using the combined disc diffusion method. Paired antibiotic discs were placed on MHA inoculated with a 0.5 McFarland bacterial suspension and incubated at 37°C overnight. An isolate was confirmed positive if either antibiotic showed an increase of ≥5 mm in zone diameter when combined with clavulanic acid compared to cephalosporin alone.

### Controls

To ensure reliability, validity, and integrity of the research data and processes, excess ﬂuid obtained during swabbing was removed by pressing and rotating the swab against the side of the tube above the level of the suspension. The plates were then inverted and placed in an incubator and incubated aerobically at 35˚C together with the positive and negative controls for about 18 hours. The *E. coli* ATCC 25922 strain was used as the positive control and negative controls involved agar plates not inoculated with the any isolates. The control and test plates were examined after an overnight incubation to ensure that the growth is confluent or near confluent. Using a ruler or Vernier caliper, the diameter of each zone of inhibition was measured in mm, on the underside of the plate.

### Data management and analysis

The data was entered into Microsoft Excel 2019, cleaned and coded then imported into Statistical Package for Social Sciences (SPSS) version 25.0 for analysis. MDR and ESBL prevalence was determined using descriptive statistics. Resistance patterns were summarized as percentages within susceptible, intermediate, and resistant categories. For inferential analysis, resistant and intermediate isolates were combined and classified as non-susceptible, while susceptible isolates remained classified as susceptible. Comparisons between milk and clinical isolates were performed using Fisher’s exact test. An overall association between isolate source (milk versus clinical) and antimicrobial non-susceptibility across all antibiotics was assessed using Fisher’s combined probability chi-square test, which combined individual p-values from antibiotic-specific Fisher’s exact tests. A p < 0.05 was regarded as statistically significant.

### Ethical considerations

Ethical approval was obtained from the School of Biomedical Sciences Research and Ethics Committee at the College of Health Science, Makerere University, Uganda (Ethical approval No.: SBS-2022-162). Written approval to carry out the study from the Clinical Microbiology Laboratory at the College of Health Sciences, Makerere was obtained from the Head of Department of Clinical Microbiology at Makerere University. Written informed consent was obtained from, owners of milk shops and diary milk supply centers and the local leaders in Kawempe Division.

## Results

**Fig 1:**
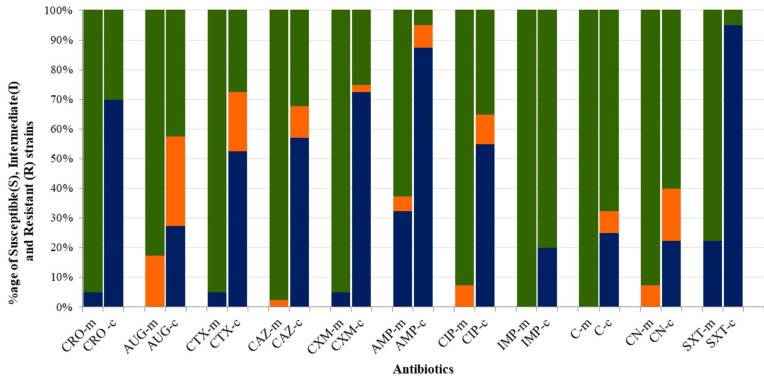
Bar graph comparing the resistance patterns of *E. coli* isolated from raw cow milk from Kawempe division, and that from clinical specimens from the clinical microbiology laboratory of Makerere University. m(R) = resistant milk isolates, m(I) = intermediate milk isolates, m(S) = sensitive milk isolates, c(R) = resistant clinical isolates, c(I) = intermediate clinical isolates, c(S) = sensitive clinical isolates.

This study assessed and evaluated the antimicrobial resistance patterns of *E. coli* isolates from raw cow milk samples obtained from Kawempe Division, Kampala, Uganda, and from clinical specimens at Makerere University, Uganda (Fig 1).

Overall, a total of 124 milk samples were collected (77 from cans and 47 from packs). 40/124 (32.26%) milk samples were contaminated with *E. coli*. Of the 32.26% of milk samples positive for *E. coli*, 31.45% were from cans and 0.81% from packs. The 40 archived *E. coli* isolates were obtained from 40 clinical specimens; blood 7/40 (17.5%), urine 18/40 (45%), sputum 4/40 (10%), vaginal swab 1/40 (2.5%), pus swab 6/40 (15%), pus aspirate 1/40 (2.5%) and pleural fluid 3/40 (7.5%) from patients aged 14–84 years.

*E. coli* isolated from raw cow milk showed highest resistance to ampicillin, AMP 13/40 (32.5%) and cotrimoxazole, SXT 9/40 (22.5%). This was followed by ceftriaxone CRO 2/40 (5%), cefotaxime CTX 2/40 (5%), and cefuroxime CXM 2/40 (5%). No resistance was observed with the rest of the antibiotics (augumentin, chloramphenicol, imipenem, gentamicin, ceftazidime, and ciprofloxacin).

*E. coli* isolated from clinical samples showed highest resistance to cotrimoxazole, SXT 38/40 (95%), ampicillin, AMP 35/40 (87.5%), cefuroxime CXM 29/40 (72.5%) and ceftriaxone CRO 28/40 (70%). This was followed by ceftazidime CAZ (57.5%), ciprofloxacin CIP (55%), and cefotaxime CTX 21/40 (52.5%). The least resistance was observed in augumentin AUG 11/40 (27.5%), chloramphenicol C 10/40 (25%), gentamicin CN 9/40 (22.5%), and imipenem IMP 8/40 (20%) (Table 2).

**Table 2:** Distribution of antimicrobial susceptibility categories among *E. coli* isolates from raw cow milk and clinical specimens.

Antibiotic	Source	Resistant, n (%)	Intermediate, n (%)	Susceptible, n (%)
**CRO**	Milk	2 (5)	0 (0)	38 (95)
	Clinical	28 (70)	0 (0)	12 (30)
**AUG**	Milk	0 (0)	7 (17.5)	33 (82.5)
	Clinical	11 (27.5)	12 (30)	17 (42.5)
**CTX**	Milk	2 (5)	0 (0)	38 (95)
	Clinical	21 (52.5)	8 (20)	11 (27.5)
**CAZ**	Milk	0 (0)	1 (2.5)	39 (97.5)
	Clinical	23 (57.5)	4 (10)	13 (32.5)
**CXM**	Milk	2 (5)	0 (0)	36 (95%)
	Clinical	29 (72.5)	1 (2.5)	10 (25)
**AMP**	Milk	13 (32.5)	2 (5)	25 (62.5)
	Clinical	35 (87.5)	3 (7.5)	2 (5)
**CIP**	Milk	0 (0)	3 (7.5)	37 (92.5)
	Clinical	22 (55)	4 (10)	14 (35)
**IMP**	Milk	0 (0)	0 (0)	40 (100)
	Clinical	8 (20)	0 (0)	20 (80)
**C**	Milk	0 (0)	0 (0)	40 (100)
	Clinical	10 (25)	3 (7.5)	27 (67.5)
**CN**	Milk	0 (0)	3 (7.5)	37 (92.5)
	Clinical	9 (22.5)	7 (17.5)	24 (60)
**SXT**	Milk	9 (22.5)	0 (0)	31 (77.5)
	Clinical	38 (95)	0 (0)	2 (5)

Antibiotics: CRO (Ceftriaxone), AUG/AMC (Augmentin/ Amoxycillin/ Clavulanic Acid), CTX (Cefotaxime), CAZ (Ceftazidime), CXM (Cefuroxime), AMP (Ampicillin), CIP (Ciprofloxacin), IMP (Imipenem), C (Chloramphenicol), CN (Gentamicin), SXT/COT (Cotrimoxazole).

The number of *E. coli* isolates from milk samples that showed resistance to at least one antibiotic was 17/40 (42.5%) with the rest 23/40 (57.5%) had either sensitive results or intermediate resistance. A total of 4 different patterns of resistance to various antibiotics was observed in *E. coli* isolates from milk samples with patterns of 1, 2 and 5 antibiotics.

Out of the 40 *E. coli* isolates obtained from the raw cow milk samples, 2 (5%) were both multidrug resistant (MDR) and positive for extended spectrum b-lactamase (ESBL); with same AMR pattern of CRO + CTX + CXM + AMP + SXT.

From the 40 *E. coli* isolates from clinical samples, 39 (97.5%) were resistant to at least 2 antibiotics. 1/40 (2.5%) *E. coli* isolate was not resistant to any of the 11 antibiotics. A total of 37 different patterns of resistance to various antibiotics was observed in *E. coli* isolates from clinical samples with patterns ranging from 2 to 10 antibiotics.

A total of 34/40 (85%) clinical *E. coli* isolates were MDR; of which 28/34 (82.4%) were ESBL positive. All MDR *E. coli* isolates from clinical samples showed cotrimoxazole (SXT) resistance with most having ampicillin (AMP), cefotaxime (CTX), ceftriaxone (CRO), cefuroxime (CXM), ciprofloxacin (CIP) resistance as well.

An overall analysis combining results across all antibiotics demonstrated a highly significant association between isolate source (raw cow milk and clinical specimens) and antimicrobial non-susceptibility. Using Fisher’s combined probability test, resistance patterns differed significantly between milk and clinical *E. coli* isolates (χ^2^ = 351.03, df = 22, p = 4.82 × 10^−61^), with clinical isolates consistently exhibiting higher levels of non-susceptibility, as shown in Table 3.

**Table 3:** Comparison of antimicrobial non-susceptibility between *E. coli* isolates from raw cow milk and clinical specimens.

Antibiotic	Milk Non-susceptible n (%)	Clinical Non-susceptible n (%)	p-value
Ceftriaxone (CRO)	2 (5.0)	28 (70.0)	1.0 × 10^−9^
Amoxicillin-clavulanate (AUG)	7 (17.5)	23 (57.5)	0.000434
Cefotaxime (CTX)	2 (5.0)	29 (72.5)	2.6 × 10^−10^
Ceftazidime (CAZ)	1(2.5)	27 (67.5)	3.3 × 10^−10^
Cefuroxime (CXM)	2 (5.0)	30 (75.0)	6.1 × 10^−11^
Ampicillin (AMP)	15 (37.5)	38 (95.0)	4.2 × 10^−8^
Ciprofloxacin (CIP)	3 (7.5)	26 (65.0)	9.2 × 10^−8^
Imipenem (IMP)	0 (0)	8 (20.0)	0.00531
Chloramphenicol (C)	0 (0)	13 (32.5)	7.6 × 10^−5^
Gentamicin (CN)	3 (7.5)	16 (40.0)	0.00120
Cotrimoxazole (SXT)	9 (22.5)	38 (95.0)	1.4 × 10^−11^

Non-susceptibility includes resistant and intermediate isolates combined. P-values were obtained using Fisher’s exact test.

(chi-square test (χ^2^) = 351.03, degrees of freedom (df) = 22, overall p-value = 4.82 × 10^−61^).

## Discussion

Antimicrobial resistance is a One Health problem that can spread between human-environment-and animal interface including animal products such as milk when consumed making it necessary to understand the patterns of resistance between milk and human/clinical samples [17].

In this study, the prevalence of *Escherichia coli* contamination in raw cow milk (32.26%) showed a good degree of milk contamination with *E. coli* and this aligned closely with values reported in some studies in Sub-Saharan Africa such as 35% in Mekelle city, Ethiopia [28]. Higher levels in Nigeria (44.1%) [23] and Zambia (51.2%) [29] reflect weaker control of contamination across the dairy value chain, while markedly lower prevalence in regulated systems, such as 6% in a study in Ethiopia [30], demonstrated the effect of enforced hygiene, pasteurization, and cold chain maintenance. These gradients indicate that contamination levels track closely with the degree of structural control within dairy systems.

The higher contamination observed in milk handled in cans (31.45%), relative to packaged milk (0.81%), points to post-milking handling as a major source of bacterial introduction and growth. Studies in Uganda and similar settings link contamination to transportation without refrigeration, prolonged storage, and reuse of inadequately sanitized containers [17,31]. These conditions support both contamination and bacterial multiplication, particularly at ambient temperatures, while repeated container use promotes biofilm formation and persistent contamination. In contrast, sealed packaging limits exposure and interrupts these pathways [13].

Resistance to ampicillin (32.5%) and cotrimoxazole (22.5%) in milk isolates falls within a moderate range relative to other settings. In Uganda, antibiotic use in livestock is widespread but largely unregulated, with bacterial diseases such as mastitis in cattle driving a sharp increase in antibiotic use often without veterinary prescription [32]. This explains increased antibiotic use of these antibiotics in diary setting such as in treatment of infections in the dairy cattle. Higher resistance to ampicillin (96.6%) and cotrimoxazole (42.1%) in a study in Tanzania [12] reflects even more intensive or less regulated antibiotic use, while values reported in Bangladesh and parts of Asia fall within a similar range to findings in this study [10,33,34]. These patterns mirror differences in antimicrobial exposure within livestock systems.

Even at moderate levels, resistance in foodborne *E. coli* has broader implications. As a commensal organism, *E. coli* acts as a reservoir for resistance genes, which can be transferred horizontally via plasmids and other mobile genetic elements [24,35]. Dairy-associated isolates therefore contribute to the wider resistome, providing a pool of resistance determinants that may later be selected and amplified in human populations.

In contrast, clinical isolates showed substantially higher resistance than milk isolates, reflecting sustained antimicrobial pressure in healthcare settings. The resistance patterns observed in clinical isolates may have been influenced by the diversity of specimen sources included in this study. Isolates derived from different infection sites are exposed to varying levels of antimicrobial pressure, which may contribute to differences in susceptibility profiles.

Resistance to cotrimoxazole (95%) and ampicillin (87.5%) in clinical isolates indicates reduced effectiveness of commonly used first-line therapies, consistent with trends reported in Uganda and other regions [20,36]. This pattern shifts treatment toward broader-spectrum antibiotics and increases selection pressure for more resistant organisms.

A key finding in this study is the high level of resistance to third-generation cephalosporins, including ceftriaxone (70%) and cefuroxime (72.5%). These antibiotics are widely used in clinical practice for the treatment of severe infections, including sepsis and complicated urinary tract infections among others [37]. Resistance at this level indicates a substantial burden of extended-spectrum β-lactamase (ESBL) producing *E. coli*. Similar resistance levels have been reported in India (72.3%) [38] and China (>70%) [34], with slightly lower levels in Zambia (52%) [5].

This pattern reflects the widespread dissemination of ESBL enzymes, which hydrolyze β-lactam antibiotics and are frequently encoded on plasmids carrying additional resistance genes [39]. As a result, resistance to cephalosporins often co-occurs with resistance to other antibiotic classes, contributing directly to multidrug resistance [40]. Clinically, this severely limits treatment options and necessitates the use of carbapenems or other last-line agents, increasing both cost and risk of further resistance selection [36].

The detection of resistance to carbapenems, particularly imipenem (20%), is therefore of critical concern in this study. Carbapenems are considered last-resort antibiotics for the treatment of infections caused by ESBL-producing organisms [41]. Resistance at this level suggests the possible emergence of carbapenemase-producing *E. coli,* which represents a significant escalation in antimicrobial resistance. Although lower than resistance to other antibiotics, this finding is epidemiologically important because carbapenem resistance is often associated with highly transmissible genetic elements that can spread rapidly within healthcare settings [42,43]. Reports from global surveillance systems indicate increasing carbapenem resistance in Enterobacteriaceae, particularly in regions with high antibiotic pressure. The presence of such resistance in this study may therefore represent an early warning signal of evolving resistance patterns [44].

The contrast in MDR prevalence between clinical isolates (85%) and milk isolates (5%) reflects the differential intensity of antimicrobial selection pressure in each setting. In Uganda, oxytetracycline was the most consumed veterinary antibacterial, with tetracyclines, aminoglycoside-penicillin combinations, and sulfonamides dominating livestock antibiotic imports from 2018 to 2020; with 97% classified as WHO veterinary critically important antimicrobials [32] largely dispensed without prescription thus creating chronic, low-intensity pressure consistent with the limited but persistent ampicillin and cotrimoxazole resistance in milk isolates. In contrast, healthcare settings in Uganda are characterized by frequent empirical use of broad-spectrum antibiotics, including third-generation cephalosporins, driving rapid co-resistance accumulation and explaining the high MDR burden in clinical isolates [36]. MDR carriage among cattle in pastoralist communities of Kasese district, Uganda was 80% and was significantly associated with MDR carriage in co-located humans [6], confirming that unregulated livestock antibiotic use has measurable cross-interface consequences. That dairy MDR can escalate sharply under intensified use is evidenced by prevalence of 57.6% in Ghana [45] and 89.4% in Egypt [46], indicating Uganda’s current lower levels may not persist without improved veterinary stewardship.

The lower ESBL prevalence in milk isolates (5%) relative to clinical isolates (70%) follows directly from the lower cumulative antibiotic burden in the dairy system; since ESBL-encoding mobile genetic elements are co-selected under sustained broad-spectrum pressure [39]. In Uganda, indiscriminate administration of antimicrobials, often in sub-optimal doses, in livestock systems has been identified as a major contributor to AMR emergence [6], yet the narrow class range used in cattle limits the breadth of co-selection compared with clinical settings. This is consistent with findings from Indonesia and Ethiopia, where substantially higher dairy MDR was accompanied by correspondingly higher ESBL prevalence of 20% and 80.8% respectively [47,48], reinforcing that ESBL emergence in dairy systems tracks MDR escalation driven by intensified antibiotic use.

Taken together, these findings show a divergence in resistance magnitude but convergence in resistance profiles between clinical and milk-derived *Escherichia coli*. There was a highly significant association between isolate source and antimicrobial resistance (p < 0.001), indicating that the observed differences are unlikely due to chance. Clinical isolates showed consistently higher non-susceptibility, reflecting strong selection pressure from frequent and broad-spectrum antibiotic use in healthcare settings.

In contrast, livestock systems involve lower but more prolonged antimicrobial exposure, which may explain the lower resistance levels in milk isolates alongside persistent resistance to commonly used antibiotics such as ampicillin and cotrimoxazole. This suggests that clinical settings drive amplification of resistance, while dairy systems act as reservoirs and potential transmission pathways, supporting a connected antimicrobial ecosystem.

Within a One Health framework, antimicrobial resistance arises from interactions across human, animal, and environmental systems. Transmission occurs through food consumption, environmental exposure, and direct contact, with mobile genetic elements such as phages, transportable DNA elements, and conjugative DNA elements which facilitate the rapid spread of resistance genes [35,49–51].

Overall, clinical settings bear the highest burden of resistance, while dairy systems contribute to its maintenance and spread. Addressing this requires coordinated action, including regulation of antibiotic use in livestock, improved hygiene in milk handling, strengthened infection prevention in healthcare, and integrated One Health surveillance.

This study had several limitations. The smaller-than-planned sample size (124 vs. 380) may have reduced statistical power and generalizability. The total number clinical samples from which the archived clinical isolates were obtained could not be ascertained with little known about the prior antimicrobial exposure pattern for the clinical isolates, hence limiting the assessment and the overall prevalence and representativeness of clinical AMR patterns. The absence of molecular typing limited assessment of genetic relatedness and resistance mechanisms. Purposive sampling may have introduced selection bias, potentially overrepresenting high-risk vendors. The cross-sectional design limited causal inference and identification of transmission pathways. Methodologically, the limited antibiotic panel and grouping of intermediate with resistant isolates reduced detail in resistance characterization. Additionally, milk and clinical isolates were not epidemiologically linked, restricting precise determination of AMR transmission between humans and cattle in Kawempe Division.

## Conclusion

This study shows a clear difference in antimicrobial resistance between clinical and milk-derived *Escherichia coli*, with clinical isolates carrying a much higher burden of resistance, MDR, and ESBL production. At the same time, the shared resistance to commonly used antibiotics like ampicillin and cotrimoxazole points to a possible overlap between human and animal reservoirs.

These findings highlight the need for context-specific antimicrobial stewardship strategies. In clinical settings, efforts should focus on rational antibiotic prescribing, strengthening diagnostic capacity, and improving infection prevention and control to reduce high resistance levels. In livestock systems, priority should be given to regulating antibiotic use, limiting non-therapeutic applications, and improving hygiene in milk production, handling, and distribution to prevent the emergence and spread of resistant bacteria.

For better conclusive findings, future studies should use larger and more representative samples, adopt longitudinal designs, and incorporate genomic approaches to better understand how resistant strains emerge and spread across the human–animal interface.

## Supporting information

S1 Table Antimicrobial resistance patterns of *E. coli* from raw cow milk. (DOCX)

S2 Table AMR patterns of *E. coli* from clinical isolates. (DOCX)

S3 Table Patterns of antimicrobial resistance phenotypes of *E. coli* strains isolated from raw cow milk. (DOCX)

S4 Table Patterns of antimicrobial resistance phenotypes of *E. coli* strains isolated from clinical specimens in the Clinical Microbiology Laboratory. (DOCX)

S5 Table Phenotypic pattern of ESBL producing *E. coli* from clinical isolates. (DOCX)

S1 Data Data. (DOCX)
