# Supplementary material for: Antimicrobial resistance patterns of Escherichia coli isolated from raw cow milk and, from clinical specimens in a tertiary institution, Uganda – A cross sectional study
Source: PLoS One. 2026 Jul 16;21(7):e0321341. doi: 10.1371/journal.pone.0321341 (PMC13375033; doi:10.1371/journal.pone.0321341)
Supplement: S2 Table — (DOCX) [file pone.0321341.s002.docx]

Supplementary Table 2. AMR patterns of *E. coli* from clinical isolates.
